# Analytic modeling and risk assessment of aerial transmission of SARS-CoV-2 virus through vaping expirations in shared micro-environments

**DOI:** 10.1007/s11356-022-20499-1

**Published:** 2022-06-27

**Authors:** Roberto A. Sussman, Eliana Golberstein, Riccardo Polosa

**Affiliations:** 1 Instituto de Ciencias Nucleares, Universidad Nacional Autónoma de México, 04510 Mexico City, Mexico; 2 Myriad Pharmaceuticals Limited, Unit 3, 36 Greenpark Rd, Penrose, 1061 Auckland New Zealand; 3 Center of Excellence for the Acceleration of HArm Reduction (CoEHAR), University of Catania, Catania, Italy

**Keywords:** SARS-CoV-2, COVID-19, Electronic cigarettes, Aerosol visibility, Risk modeling

## Abstract

It is well known that airborne transmission of COVID-19 in indoor spaces occurs through various respiratory activities: breathing, vocalizing, coughing, and sneezing. However, there is a complete lack of knowledge of its possible transmission through exhalations of e-cigarette aerosol (ECA), which is also a respiratory activity. E-cigarettes have become widely popular among smokers seeking a much safer way of nicotine consumption than smoking. Due to restrictive lockdown measures taken during the COVID-19 pandemic, many smokers and vapers (e-cigarette users) were confined to shared indoor spaces, making it necessary to assess the risk of SARS-CoV-2 virus aerial transmission through their exhalations. We summarize inferred knowledge of respiratory particles emission and transport through ECA, as well as a theoretical framework for explaining the visibility of exhaled ECA, which has safety implications and is absent in other respiratory activities (apart from smoking). We also summarize and briefly discuss the effects of new SARS-CoV-2 variants, vaccination rates, and environmental factors that may influence the spread of COVID-19. To estimate the risk of SARS-CoV-2 virus aerial transmission associated with vaping exhalations, we adapt a theoretical risk model that has been used to analyze the risks associated with other respiratory activities in shared indoor spaces. We consider home and restaurant scenarios, with natural and mechanical ventilation, with occupants wearing and not wearing face masks. We consider as “control case” or baseline risk scenario an indoor space (home and restaurant) where respiratory droplets and droplet nuclei are uniformly distributed and aerial contagion risk might originate exclusively from occupants exclusively rest breathing, assuming this to be the only (unavoidable) respiratory activity they all carry on. If an infected occupant uses an e-cigarette in a home or restaurant scenarios, bystanders not wearing face masks exposed to the resulting ECA expirations face a $1\,\%$ increase of risk of contagion with respect the control case. This relative added risk with respect to the control case becomes $5-17 \%$ for high-intensity vaping, $44-176 \%$, and over $260 \%$ for speaking for various periods or coughing (all without vaping). Infectious emissions are significantly modified by mechanical ventilation, face mask usage, vaccination, and environmental factors, but given the lack of empiric evidence, we assume as a working hypothesis that all basic parameters of respiratory activities are equally (or roughly equally) affected by these factors. Hence, the relative risk percentages with respect to the control state should remain roughly the same under a wide range of varying conditions. By avoiding direct exposure to the visible exhaled vaping jet, wearers of commonly used face masks are well protected from respiratory droplets and droplet nuclei directly emitted by mask-less vapers. Compared to the control case of an already existing (unavoidable) risk from continuous breathing, vaping emissions in shared indoor spaces pose just a negligible additional risk of COVID-19 contagion. We consider that it is not necessary to take additional preventive measures beyond those already prescribed (1.5 m separation and wearing face masks) in order to protect bystanders from this contagion.

## Introduction

The most familiar respiratory activities, breathing, vocalizing, coughing, and sneezing, have been shown to transmit COVID-19; however, there is no data or direct evidence of this transmission through the aerosol exhaled by vapers (e-cigarette users). Nevertheless, it cannot be ruled out that vaping (as a respiratory activity like smoking) can plausibly transmit COVID-19 through its environmental exhalations. The current COVID-19 pandemic is a very complex and disruptive event involving many factors besides the necessary medical response. This has forced governments to implement prevention and containment measures affecting millions of individuals, including vapers and smokers finding themselves confined (or forced to spend much time) in shared indoor spaces. Thus, evaluating theoretically the risks involved in this possible contagion route is crucial. As a result of the present study, we provide a self-consistent risk model of COVID-19 contagion while using this respiratory route in shared indoor environments, considering factors such as visibility, ventilation, mask usage, SARS-CoV-2 variants, vaccination rates, and environmental factors. We undertake a risk assessment of COVID-19 transmission in shared indoor spaces from exhaled aerosols in two settings: a home and a restaurant with natural and mechanical ventilation.

The present paper is based on the adaptation to vaping exhalations in a stable environment of the dose-response exponential risk model (Watanabe et al. 2010; Sze To and Chao 2010) that has been considered mostly for the evaluation of outbreaks of COVID-19 transmission risk in shared indoor spaces, taking into consideration various respiratory activities and environmental parameters (see authoritative review with numerous references in Peng et al. (2021)). As explained in this review, a common set of assumptions in this type of modeling is to consider the “shared space airborne” scenario with individuals sharing air in the same room, all exposed to an infected person in an indoor space regarded as a “box” in which respiratory droplets and their nuclei (known as “aerosols”) have been uniformly distributed throughout the full indoor volume (we consider a home and a restaurant scenarios). In particular, among the risk evaluation studies reviewed in Peng et al. (2021), we adapted to vaping exhalations, a simplified and modified version of the exponential dose-response reaction model developed by Buonanno et al. (2020a, 2020b) (hereafter BMS), based on the notion of “infective quanta” (the virus concentration needed to infect 63% of exposed individuals) constructed with data on SARS-CoV-2 infective parameters that was available by the middle of 2020.

Given the lack of previous studies of vaping as a respiratory activity and thus absence of empiric evidence of the type of respiratory particles (droplets or droplet nuclei numbers and diameters) potentially transmitted through exhaled ECA, we inferred this data in a previous paper (see Sussman et al. (2021b)), using cigarette smoking and mouth breathing as useful proxies for the respiratory mechanics and droplet emission that should occur, with transmission distances computed by modeling ECA as an intermittent turbulent jet evolving into a puff. These findings have been incorporated in our adaptation of the BMS risk model to compute the viral quanta emissions that should correspond to exhaled ECA to be able to compare them with those of vocalizing and coughing, all compared with respect to a baseline “control state” defined by the unavoidable respiratory particle transmission from exclusive continuous breathing. As we show in the “Discussion I: comparison with results of BMS” section, our approach is consistent with that of BMS, but the interpretation of outcomes is different.

Since wearing face masks has become a universally accepted preventive measure, we discuss the risk modifications involved for masked bystanders exposed to emissions from potentially infected unmasked individuals, as vapers must (at least) momentarily remove their face masks in order to vape. However, the complexity of COVID-19 as an evolving pandemic requires considering other important factors besides face mask wearing, such as SARS-CoV-2 variants and vaccination, as well as environmental factors (whose effects are a more indirect and involve a higher level of speculation). Evidently, as we argue in the “Background III: viral variants, vaccination, and environmental factors” section, all these factors may modify contagion risks. To address this complexity, we assume (see the “Discussion III: how robust is our relative risk model?” section) as a working hypothesis that all respiratory activities are equally affected by modifications from these factors (given the absence of empiric data on this issue). As a consequence, regardless of the modifications of absolute risks for every respiratory activity, the relative risks referred should remain stable and roughly unchanged with respect to the control state of exclusive breathing.

An important property that distinguishes ECA exhalations from other respiratory activities (save smoking) is visibility, which emerges from the optical properties of ECA droplets (light scattering Ruzer and Harley 2012). Visibility has significant psychological and safety implications: it allows those surrounding potentially infectious vapers to instinctively place themselves away from the area of direct exposure clearly delineated by the visible exhaled cloud (something impossible to do with expirations from other respiratory activities, except smoking, see the “Background II: visualization of the respiratory flow through ECA” section).

Vaping as a substitute of cigarette smoking is part of a tobacco harm reduction strategy that empowers users and significantly decreases the harm caused by smoking. However, in an effort to combat the current COVID-19 pandemic, health authorities throughout the world have implemented various containment measures, including curtailing social activities and home confinement recommendations that have forced millions of vapers to share indoor spaces with those who do not smoke or vape. Once under confinement, smokers who switched to vaping perceive that their decision to switch to vaping can enhance harm reduction efforts in an important way with respect to those who continue smoking. First, environmental ECA exposes bystanders to a minuscule amount of hazardous compounds in comparison with ETS. Secondly, the time and spatial scope of exposure is very limited, as environmental ECA droplets rapidly evaporate into a fast dispersing gas phase, practically making ECA imperceptible in a time frame below 1 min and 2 m or less from the vaper. As a contrast, ETS exposes bystanders to a significantly larger toxicant charge in whole rooms and for extended periods: up to 40 min per cigarette (see comparison between environmental ECA and ETS in Liu et al. (2017); Zhao et al. (2017); Martuzevicius et al. (2019); Palmisani et al. (2019)). As a consequence, smokers who switch to vaping enhance the safety of those with whom they share an indoor space under confinement.

It is important to note that this article only discusses the risk of potential SARS-CoV-2 transmission by exhaled ECA, not with risks of COVID-19 contamination or illness of vapers due to possible effects on the respiratory system of vaping, or other possible health hazards caused by exposure to inhaled or exhaled vapors derived from the use of e-cigarettes to substitute tobacco smoking. As reference on these issues, we recommend readers to consult the short review of the literature that we provide in Sussman et al. (2021a), as well as earlier work on the relative safety of e-cigarettes with respect to smoking (Farsalinos and Polosa (2014); RCP (2016); McNeill et al. (2018); NASEM (2018); and Polosa et al. (2019)).

As a further disclaimer, we will not address the risk of COVID-19 spreading through respiratory droplets transmitted by environmental tobacco smoke (ETS), although our risk assessment of low-intensity vaping (practiced by 80 to 90% of vapers by puffing with mouth retention) substantially differs from ETS by the complete lack of side stream emissions (originating from the smoker from the burning/smoldering tip of the cigarette that does not enter the respiratory system). However, we emphasize that exposure to ETS indoors is more hazardous than exposure to exhaled ECA in every way except, possibly, for transmission of the SARS-CoV-2 virus.

**Fig. 1:**
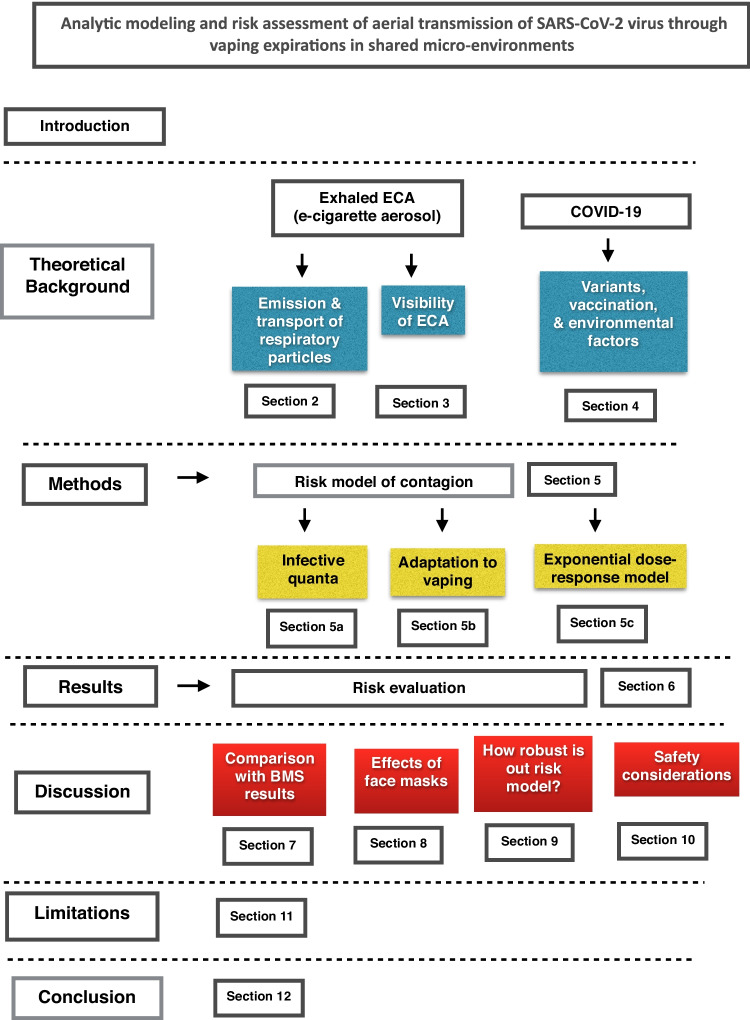
Methodological structure. See further explanation in the final paragraph of the “Introduction”

The methodological structure of the paper is displayed graphically by the diagram of Fig. 1. In what follows, we complement the information provided in this diagram. Theoretical background material is presented in three sections. Two of these sections concern properties of ECA relevant to COVID-19 transmission: the “Background I: emission and transport of respiratory particles by ECA” section provides a brief summary of results of Sussman et al. (2021b) on emission and transport of respiratory particles (droplets and nuclei), while the “Background II: visualization of the respiratory flow through ECA” section examines the visibility of exhaled ECA, commenting on its safety implications in the “IV: safety considerations” section. The third one is the “Background III: viral variants, vaccination, and environmental factors” section, presenting a summary of various factors affecting COVID-19 evolution, mainly the new variants of the virus named with Greek letters, the immunity protection through various vaccines, but also environmental factors, such as air pollution and atmospheric stability that may influence the virus propagation. We present in the “Methods: risk model of contagion” section a risk model of SARS-CoV-2 contagion in shared indoor spaces (home and restaurant scenarios, natural and mechanical ventilation) based on an adaptation and simplification of the model proposed by BMS (Buonanno et al. 2020a, b). Results of the application of the above described risk model are presented together with an extensive discussion in the “Results: risk evaluation” section. A complete discussion of these results is given in four sections: a model validation by consistency with the results found by BMS in one scenario and on the Guangzhou restaurant in the “Discussion I: comparison with results of BMS” section, a full discussion is provided in the “Discussion II: effects of face mask wearing” section on the effect of face mask wearing, since the risk model was developed without considering this preventive measure, though face masks are seldom worn in a home scenario and compliance with this measure is lax in restaurant scenarios. In the “Discussion III: how robust is our relative risk model?” section, we provide a brief discussion of the validity and robustness of our model in view of effects of SARS-CoV-2 variants and vaccination, as well as environmental factors, while a discussion of safety issues is given in the “IV: safety considerations” section. We show that relative risks are stable under the modification of absolute risks by these factors. The limitations and our conclusion are presented in the “Limitations” and “Conclusion” sections.

## Background I: emission and transport of respiratory particles by ECA

### Respiratory particles emission

In order to evaluate infection risks from vaping expirations in shared indoor spaces, we need empiric data on relevant parameters of these expirations: tidal expired volume and characteristics of exhaled ECA carrying respiratory droplets, droplet emissions in such expirations, and distances along which these droplets should be transported by them. As we argued in in Sussman et al. (2021b), the lack of this data requires its inference through theoretical modeling guided by phenomena (on which this data exists) that can serve as proxies for vaping exhalations. Using this inferred outcome droplet propagation, distances can be estimated through a model of a turbulent intermittent jet evolving into a puff.

In what follows, we summarize the main results of the theoretical modeling undertaken in Sussman et al. (2021b):

- **“Mouth to Lung” (MTL) low-intensity vaping and smoking.** The outcomes displayed in Table SM(2) and Table 2 of Sussman et al. (2021b) suggest mean expired tidal volumes of $V_T=700-900 \text {cm}^3$ potentially carrying $N_p=6 - 210$ respiratory droplets per exhalation (median $N_p=$ 39.9, deviation 67.3), overwhelmingly in the submicron range (typically peaking at $d_p=0.3-0.8 \mu \text {m}$) and droplet number densities well below $n_p = 1 \text {cm}^{-3}$. As shown in Figure SM(2) of Sussman et al. (2021b), this is the style of vaping involving low-powered devices practiced by 80–90% of vapers in the main consumer markets (the USA and the UK). However, the proxies we have used (cigarette smoking and mouth breathing) exhibit a wide individual variation in expired volumes, puffing parameters, and droplet emission, all of which should occur also in vaping. Thus, the inferred data we have mentioned excludes the small minority of outlier individuals known as “super emitters” possibly emitting as much as $N_p\sim 1000$ respiratory droplets per exhalation in expired tidal volumes of up to 2 LT.
- **“Direct to Lung” (DTL) high-intensity vaping.** It involves high-powered tank devices that allow for a wider spectrum of deeper respiratory intensity than MTL vaping. It should involve a higher rate of droplet emission and expired volumes of 2–3 LT. Perhaps the closest analogue to infer its droplet emission rate among the studies listed in Table 2 of Sussman et al. (2021b) is breathing at fractional residual capacity in Almstrand et al. (2010) that reported emission rates of around 1000/LT. However, this style of vaping is practiced by a small minority of vapers (roughly 10–20%), with its upper end being extreme vaping (the so-called cloud chasers) that is only practiced in competitions or exhibitions. Evidently, this type of extreme vaping cannot be sustained for long periods and is not representative even of DTL vapers.

While the inferred droplet numbers in the upper end of high-intensity DTL vaping can be comparable with low end numbers for vocalizing, the latter involves modes with larger mean diameters because of distinct droplet generation processes (Asadi et al. 2019; Morawska et al. 2009; Johnson et al. 2011).

### Distance for direct exposure

The distance range that vaping expirations can transport respiratory droplets provides the spatial scope of direct pathogen exposure through exhaled ECA, modeled in Sussman et al. (2021b) as an intermittent jet evolving as the exhalation ends into a turbulent puff. The scope of direct exposure is the displacement or penetration distance of the jet in the direction of the momentum of the jet at exhalation. The parameters characterizing the jet are expired tidal volume of air diluted ECA mentioned in the “Respiratory particles emission” section and exhalation centerline velocities estimated in Sussman et al. (2021b) as $U_0=0.5-3$ m/s (MTL vaping) and $U_0=1,5-5$ m/s (DTL vaping), which (assuming horizontal exhalation) yields a distance spread of 0.5–2.0 m for the MTL vaping and over 2.0 m for DTL. The maximal penetration goes beyond that afforded by the momentum trust of the starting jet, with the puff further evolving at lesser speeds. Before the puff stage, centerline velocities drop to about 0.2 m/s at different times and distances when fluid injection stops in all cases.

Given its short time duration and close distance scope of the momentum trusted staring jet, the analytic model analyzed in Sussman et al. (2021b) provides a reasonably good inference of the distance and direction that bystanders should keep to minimize the risk of direct exposure. As the jet evolves, it mixes with surrounding air, with entrained air reaching about 40% of the jet mass as exhalation (fluid injection) ends at the transition towards the puff regime (Ghaem-Maghami 2006; Ghaem-Maghami and Johari 2010). Since at this point the jet velocities become comparable to typical velocities of $\sim 10$ cm/s (and up to 25 cm/s) of airflow currents in home environments, even in still air with natural ventilation (Matthews et al. 1989; Berlanga et al. 2017), the puff can be easily destabilized by vortex motion generated through turbulent mixing from the large velocity fluctuations produced by the entrainment (Wei and Li 2015; Vuorinen et al. 2020).

Once the puff becomes disrupted, bystanders face indirect exposure to mostly droplet nuclei, as submicron respiratory droplets evaporate almost instantly as they are exhaled (see Nicas et al. 2005). Turbulence and thermal buoyancy and stratification become important factors when the jet initial momentum decreases, more so when the vaper (the source) moves or walks (Wang and Chow 2011). Mechanical ventilation (mixed or displaced) He et al. (2011); Gao and Niu (2007); Gao et al. (2008) produces a faster disruption and dispersion of the slow moving puff through their own turbulent, thermal stratification and droplet dispersion patterns. In general, the dispersed submicron droplets and droplet nuclei can remain buoyant for hours, with mixing ventilation tending to uniformly spread them, whereas directed ventilation tends to stratify them along different temperature layers. The detailed description of droplet dispersion after the puff disruption is a complicated process that requires computational techniques that are beyond the scope of this paper (see comprehensive analysis in Vuorinen et al. (2020)).

## Background II: visualization of the respiratory flow through ECA

As shown in Sussman et al. (2021b), respiratory droplets that would be carried by exhaled ECA should be overwhelmingly submicron (just as ECA droplets). As a consequence, ECA droplets and the much fewer respiratory droplets accompanying them should follow the fluid flow of ECA approximately as molecular contaminants, thus acting like visual tracers of the expiratory flow (Ai et al. 2020; Nazaroff 2004). This visibility is shared with smoking (see Gupta et al. 2009, 2010; Ivanov 2019), but is absent in other respiratory expirations (breathing, vocalizing, coughing, sneezing). It has important psychological and safety implications, since bystanders can instinctively detect (and avoid) the area of direct exposure. We provide here a brief discussion of optical properties of aerosols that allow for their visualization (see comprehensive explanation in Ruzer and Harley (2012); Hinds (1999); Kulkarni and K (2011)).

Visualization and coloring of aerosols follow from the interaction of light with its particulate phase through absorption and scattering (refraction, reflection, and diffraction), which depends on the particles’ number density $n_p$, chemical nature, and the ratio of their diameters to visible light wavelengths: $\alpha =\pi d_p/\lambda$ with $\lambda =0.4-0.7 \mu \text {m}$. This interaction is described in relatively simple terms for ultra-fine particles ($d_p< 0.05 \mu \text {m}$ or $\alpha \ll 1$) by Rayleigh’s molecular scattering theory and for large particles with $d_p> 100 \mu \text {m}$ in terms of geometric optics. Particles with diameters in the intermediate range correspond to Mie scattering theory, which becomes particularly complicated for $d_p$ roughly comparable to $\lambda$ or $\alpha \approx \pi$, as is the case for ECA and respiratory droplets. Since the latter are liquid droplets, they are in practice non-absorbing so that scattering is the dominant effect.

A simpler approach to aerosol optics follows from the notion of light extinction, the loss of intensity *I* from absorption, and scattering in the direction of an incident parallel non-polarized light beam, with intensity $I_0$ and cross section distance *L* within an aerosol. This is described by the extinction coefficient $\sigma _e$ through the Lambert-Beer (or Bouguer) law

1 $$\begin{aligned} I = I_0 \text {e}^{-\sigma _e L},\qquad \sigma _e = \frac{\pi }{4}Q_e n \bar{d}^2, \end{aligned}$$

where *n* is the total particle number density, $\bar{d}$ is the mean particle diameter, and, for non-absorbing particles, $Q_e\approx Q_{\text {scatt}}$ is the scattering extinction efficiency taken as constant (a valid approximation for a fixed $\lambda$ and a very small diameter range around $\bar{d}$).

The fact that *n* in non-biological aerosols (like exhaled ECA) is much larger than in bioaerosols that are just “airborne” without ECA (as in “normal” respiratory activities) explains why exhaled ECA is visible while the bioaerosols are not. To illustrate this point quantitatively, consider the light extinction law Eq. 1 for an incident beam with wavelength $\lambda =0.5 \mu \text {m}$ crossing an exhaled ECA jet (see figure 2 of Sussman et al. 2021b). From the mean diameters obtained in Sussman et al. (2021b), we have $\bar{d}= \lambda = 0.5 \mu \text {m}$ and $\bar{d}= 0.4 \mu \text {m}$ for respiratory and ECA droplets (the latter just at exhalation before their rapid evaporation), while (from figure 16.2 of Hinds 1999) we have $Q_e= 2, 3.5$ respectively for ECA and respiratory droplets, as their respective refractive indices are roughly those of water and VG: $m=1.33, 1.5$. While *L* is the same (same fluid jet) and $\bar{d}$ and $Q_e$ have comparable values for both types of droplets, the large difference in *n* makes a significant effect in light transmission through the beam that indicates the aerosol visibility in its specific direction if $I/I_0< 1$. In numbers, we have $n_p\sim 10^7 \text {cm}^{-3}$ and $L=15 \text {cm}$ for ECA just at exhalation, leading to impaired light transmission, $I/I_0=0.51$, while at 1 m distance after significant dilution: $\bar{d}\sim 0.1, L=25 \text {cm}$ and $n_p\sim 10^5 \text {cm}^{-3}$, we have almost full light transmission $I/I_0=0.99$. For respiratory droplets $n_p\sim 0.1-1 \text {cm}^{-3}$, leading to practically full light transmission $I/I_0\approx 1$ irrespective of *L* (in the appropriate ranges delineated by the exhaled jet).

Extinction through a parallel beam provides a limited description of light scattering in an aerosol, which occurs through each droplet in all directions. The rigorous description through Mie scattering theory is beyond the scope of this paper, but it is evident that droplet numbers play a significant role since total scattered intensity is basically the sum of scattered intensity from each droplet. The light extinction law Eq. 1 is valid under the assumption that photons in all directions follow classical paths and their scattering complies with a Poisson distribution (a good approximation for sufficiently diluted aerosols). Under these assumptions, the mean free path between scattering events is simply (see chapter 13 of Kulkarni and K 2011) $\ell = 1/\sigma _e=1/(C_e n)$, where $C_e\propto \bar{d}^2$ is the extinction cross section per droplet. Evidently, light scattering in the scales of the ECA jet is negligible for bioaerosols with very low *n*, as the mean free path $\ell$ becomes extremely large (much larger than the bioaerosol scale). For $\bar{d}= 0.5 \mu \text {m}, Q_e=2$ and $n\sim 0.1-1 \text {cm}^{-3}$, we have $\ell \rightarrow 10^8-10^9 \text {cm}=10^3-10^4 \text {km}$, a huge value that explains the practical lack of scattering events in these bioaerosols.

## Background III: viral variants, vaccination, and environmental factors

### SARS-CoV-2 variants and vaccines

As of January 19, 2022, more than 9.5 billion COVID-19 vaccine doses have been administered globally (WHO 2022), and several evidence demonstrated vaccination safety and effectiveness in reducing COVID-19 burden (Olariu et al. (2021); Ferrara et al. (2022)). However, the simultaneous emergence of SARS-CoV-2 variants is a major challenge to the current global vaccination campaign, mostly driven by their higher transmissibility and the possible reduction of neutralizing activity of vaccine-induced antibodies towards novel viral strains (Ferrara et al. (2022); Mistry et al. (2021)), particularly the B.1.1.529 variant of concern: Omicron (Dolgin (2022)). Preliminary data observed that COVID-19 vaccines maintain relative effectiveness against severe to moderate disease sustained by the circulating variants (Mistry et al. 2021), but faster vaccination provision and booster immunization have proved to restore a significant level of protection and are helping control the pandemic.

The further emergence and spread of variants are an expected part of the evolution of SARS-CoV-2. Surveillance systems observed that some antigenically distinct SARS-CoV-2 strains — classified as Variants of Concern (VOC) — exhibit a substantial growth advantage over the ancestral SARS-CoV-2 strain (wild type) and other VOCs (Hsu et al. (2022)).

Modeling the full extent to which a VOC could infect individuals and/or evade existing vaccine- or infection-derived immunity is challenged by several factors, including computation approaches, changes in community mitigation strategies, vaccination implementation and policies, and exposition to different SARS-CoV-2 variants across the previous pandemic waves (Saxena et al. (2021)). However, epidemiological data allow at estimating proxy measures of infectiveness dependence of new variants, particularly those which had a major impact on COVID-19 burden, such as B.1.1.7 (Alpha), B.1.617.2 (Delta), and B.1.1.529 (Omicron) VOCs.

The Alpha variant was the first highly impacting VOC to be detected in the UK in December 2020, and reported to be 50% more contagious than wild-type SARS-CoV-2 strain from Wuhan: Yang and Shaman (2021). Estimates of the increased transmissibility found Delta variant to be twice as contagious as the original SARS-CoV-2 strain and around 40 to 60% more transmissible than Alpha (SPIMO (2021)). Omicron variant, firstly detected in South Africa in late 2021, was found to be up to 4 times as infectious as the Delta variant among vaccinated people, and has rapidly become the dominant variant in the vast majority of countries (Lyngse et al. (2021)). More recent (though speculative) information on the Omicron variant can be found in Koslow (2022) and Maxmen (2021).

### Environmental factors

As a disease whose primary cause is overwhelmingly characterized by airborne viral transmission (Domingo et al. 2020), there is an abundant literature on numerous factors acting as secondary effects connected with the physical properties of air that might contribute to enhance or decrease this airborne transmission. In particular, it is well known from previous pandemics that variations of temperature and humidity do affect viral transmission, as low temperatures and humidity allow for respiratory droplets of larger size (and thus with possible higher viral load) to remain more time in the environment and travel longer distances before evaporating into smaller and solid nuclei (Nicas et al. (2005); Diao et al. (2021)). Other factors that might affect contagion rates, originating in outdoors but with possible indoor influence, are air pollution and atmospheric dynamics. These topics have become research subjects of interest, though existing studies are still limited in scope and methodology (see comprehensive literature reviews in Bourdrel et al. (2021); Brunekreef et al. (2021)). As all authors recognize, research on environmental effects on viral transmission is still in its infancy, but there is a pressing need to undertake it for guiding and improving a multidisciplinary approach to enhance as much as possible institutional readiness in future pandemics (Coccia 2021c, 2020, 2022). We provide below a brief discussion of these topics.

#### Outdoor vs indoor exposure

There is a widespread consensus that infected subjects in outdoor spaces represent a negligible proportion with respect to those infected indoors, a consensus based at least on data that includes VOCs before the emergence of the highly contagions omicron variant (see comprehensive review of studies in Dias and Tchepel (2018) and a more recent one during the pandemic in Bulfone et al. (2020) and critical comments in Contini and Costabile (2020)). There is a noticeable heterogeneity of risk estimates, but all reviewed studies based on actual data reviewed in the above cited review report low ratios of outdoor contagion risk, as low as 1/1000 with respect to indoor contagion. Although a large share of indoor PM originates outdoors (40–70%, depending on the degree of insulation of indoor spaces: housing facilities, offices, and business, see Chapter 6 of Ruzer and Harley (2012)), There is a complicated relation (mediated by anthropogenic factors) between the type and chemical composition of PM in outdoors and indoor spaces, including micro-environments (Schweizer et al. 2007).

However, while the significant decrease in risk of outdoor exposure to the SARS-CoV-2 virus is accepted, all studies find it extremely difficult to overcome the the near impossibility to determine with minimal certainty if the reported contagions actually happened outdoors, as people (even those working outdoors) normally do spend a non-negligible portion of their time indoors (Schweizer et al. 2007). Individuals are often unaware and subjective when asked when and where infections occurred. The prevailing uncertainty is further complicated by the fact that the probability of contagion might be much higher in some outdoor spaces with high occupancy, such as crowded restaurant terraces, outdoor markets, sports stadiums, or political demonstrations, events in which it is practically impossible to obtain even inferential risk estimates. Nevertheless, as we argue in the “Discussion III: how robust is our relative risk model?” section, our risk model is concerned with stable indoor scenarios and thus the complexity of outdoor conditions should bear little influence on the main results.

#### Air pollution

A large body of literature with diverse content and quality has emerged trying to find links between levels of air pollution and the spread of COVID-19 at regional and even national level. Two comprehensive reviews of this literature up to November 2020 are found in Brunekreef et al. (2021) and Walton (2021). We describe below the mechanisms with which air pollution can affect COVID-19 transmission:

- SARS-CoV-2 virus transports through air pollution particulates $\text {PM}_{10}, \text {PM}_{2.5}$ or even hyperfine PM. This type of transmission remains very speculative and very likely an extremely remote possibility (Dias and Tchepel 2018; Brunekreef et al. 2021; Walton 2021). Air pollution typically originates in open outdoor combustion sources: motor vehicles and a wide variety of sources from industrial activity. It can also arise from natural causes (dust and other crustal phenomena), all of which spreading to extremely large dispersion volumes. The same comments stated before on the complicated relation between outdoor and indoor spaces and sources discussed extensively in Chapter 6 of Ruzer and Harley (2012) and Schweizer et al. (2007) apply to air pollution, implying a much weaker effect of penetration of air outdoor pollution into indoor spaces (pending on the degree of insulation) where contracting COVID-19 is easier and most frequent. Moreover, the relative number densities make it extremely difficult to regard the transport of viable virus through air pollution a realistic possibility. PM density numbers in polluted areas can be up to 8 orders of magnitude larger than that of respiratory particles from all respiratory activities (Contini and Costabile 2020). Also, viable SARS-CoV-2 in aerosol form seems to have a limited lifetime measures in hours (Van Doremalen et al. (2020); Fears et al. (2020)). Therefore, even if we assume viral transport following an outdoor to indoor path to be possible (an adventurous guess given the toxic potential of constituents of air pollution: not only PM, but also its gaseous phase, Contini and Costabile 2020), the probability of pollution PM loaded with viable virus entering an indoor space is negligible. Notice that this type of indoor contagion originating outdoors is a very different situation (involving much larger space volume) from that of transport within flats in buildings in a Hong Kong housing complex during the fist SARS pandemic (McKinney et al. 2006).
- Biological mechanisms have been identified that account for a link between air pollution (PM and gasses $\text {SO}_2$, CO, $\text {NO}_2$, and $\text {O}_3$) and respiratory and/or cardiovascular disease (Lelieveld et al. 2015), which are related to comorbidities representing major risk factors that increase susceptibility for contagion and evolution towards a more severe COVID-19 stage. Many highly polluted areas are characterized by higher frequency of personal contacts, mostly business but also social in shared hospitality venues and often the permanent residents belong to lower socioeconomic status (see Contini and Costabile 2020). Even if wearing face masks, the potential for non-COVID disease is high considering their limitations discussed in the “Discussion II: effects of face mask wearing” section. Most studies are of the ecological type that correlate short and long term exposure to averaged air pollution measurements reported in jurisdictions ranging from metropolitan areas to districts and provinces within specific countries and even comparing results from countries. As examples of ecological type of studies, we have major urban areas in China (Zheng et al. (2021)), county-level study covering the USA (Wu et al. (2020)), Italy (Conticini et al. (2020); Coccia (2021a); Fattorini and Regoli (2020)), Poland (Semczuk-Kaczmarek et al. (2021)), 33 European countries (Lembo et al. (2021)), and OCDE countries (Barnett-Itzhaki and Levi (2021)). Evidently, country-wide studies offer a very coarse and limited capacity for the control of confounders.

These ecological studies face (specially if they are short term) many uncertainties and major difficulties to disentangle an independent effect from air pollution by controlling for the many confounders due to the differences between jurisdictions in other factors, such as socioeconomic development, mobility of population, and climate that might favor thermal inversions, not to mention the fact that many individuals with mild symptoms or asymptomatic are not registered in contact tracings as COVID-19 contagions. All these factors make it is practically impossible to estimate the residual confounding (see Riccò et al. 2020 ).

Another complication arises from the fact that the constituents of air pollution exhibit significant time and space variation at municipal, regional, and national level (Fatimah (2016); Hayes et al. (2013); Crippa et al. (2013)). In particular, in the context of COVID-19, inconsistent results (mostly on the abundance of pollutant species allegedly contributing to infection rates) were observed in a comparison of ecological studies from four countries: Canada, Italy, England, and the USA (Huang et al. (2021)). Ecological studies studies might become more useful if complemented and contrasted with an epidemiological approach based on individual registries of COVID-19 cases, admissions to hospitals, and mortality, though in many jurisdictions and countries such data is either absent or scarce or incomplete (Contini and Costabile (2020)) (see also extensive discussion of this approach in Walton (2021)). There is also a deeper theoretical critique: regression models (in the ecological approach) are not appropriate for epidemiological research, instead studies must be based on testing predictive generalizations against data. In other words, science should be complemented (not substituted) by regression techniques (Cox Jr and Popken (2020)).

#### Atmospheric dynamics and stability

Another environmental factor that might be possibly related to the spread of COVID-19 is atmospheric stability characterized by slow stationary wind patterns, as such conditions might increase or decrease the propagation of airborne virus loaded respiratory particles, either by changes in temperature or humidity and also in population mobility (Hassan et al. 2021; Bhaganagar and Bhimireddy 2020). The fact that COVID-19 had such an alarming outbreak in Northern Italy and Central Spain motivated research on the possible existence special meteorological conditions in these jurisdictions (Sanchez-Lorenzo et al. 2021; Coccia 2021a, b). However, research of climatological effects on the spread of COVID-19 faces similar methodological problems as those looking at effects from air pollution: basically, the need to regard these effects from a multidisciplinary point of view as summarized and discussed before.

## Methods: risk model of contagion

Having considered the inferred data obtained in Sussman et al. (2021b) on exhaled tidal volumes, emission rates, type of respiratory droplets, and exhalation distance spreads, as well as the visibility of these exhalations, we need to evaluate exposure risks of bystanders sharing indoor spaces with infected vapers within the “box” model of a given number of occupants sharing a common indoor space as described in the review (Peng et al. 2021). However, as opposed to the approach and most examples contained in this review, we do not seek to describe the conditions for infection outbreaks, but conditions describing a relatively stable environment that can be be modeled by taking into account exposure to SARS-CoV-2 viruses potentially carried by a total mass of droplet emission already present in indoor spaces irrespective of whether the exposure is direct or indirect. Still, by using the parameters characterizing the infection outbreak in the famous Guangzhou restaurant, we are able in the “Discussion I: comparison with results of BMS” section to provide a good approximation to the estimation of density of infective quanta and infection probability that was estimated by BMS for this event.

However, given the visibility of vaping (and smoking) exhalations, it is safe to assume that bystanders sharing indoor spaces with vapers will, extremely likely, avoid direct exposure to the exhaled jet and thus will be mostly subjected to indirect exposure to (mostly) droplet nuclei dispersed by surrounding air currents once the jet has become a puff subjected to thermal buoyancy and turbulent mixing. It is also important for the consistent risk model that we require to examine viral exposure of a total mass of droplet emission for other expiratory activities (breathing, vocalizing, coughing, sneezing) under the same assumptions, though for these activities avoidance of direct exposure is not instinctive because the exhalations are not visible.

The data inferred in Sussman et al. (2021b) considered generic respiratory particles (droplets and droplet nuclei) without reference to a specific pathogen/disease and have not evaluated infection risks of exposed susceptible individuals. We undertake now this evaluation, referring specifically to the available information on the parameters of the SARS-CoV-2 virus, assuming as well that emitted respiratory droplets or droplet nuclei potentially carrying this virus have been dispersed uniformly throughout a given indoor micro-environment.

**Fig. 2:**
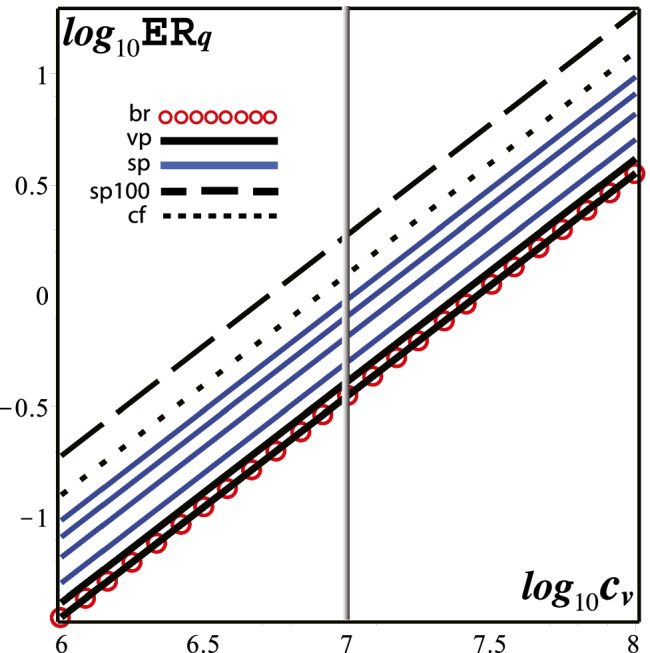
Quanta emission rates. The curves display $\texttt {ER}_q$ (quanta/hour) as a function of viral load $c_v$ (RNA copies/mL) for various expiratory activities: rest breathing (br), low- and high-intensity vaping (vp), speaking (bottom to top) $10, 20, 30, 40 \%$ of the hour (sp), coughing (cf), and speaking $100 \%$ of the time (sp100). Numerical values of ${\texttt {ER}}_q$ for $c_v=10^7$ RNA copies/mL (vertical line) are listed and discussed in the text. The ratios between these activities and rest breathing (taken as the case control scenario) are displayed in Fig. 5

Besides visibility, another extremely important feature that fully characterizes exposure risks from vaping expirations is the significant shortening of exposure time because of their intermittent and episodic nature: an infectious vaper (symptomatic or not) would emit respiratory droplets only while vaping (120–200 daily exhalations, Dautzenberg and Bricard (2015); Cahours and Prasad (2018)), whereas the same vaper will emit respiratory droplets continuously just by normal rest breathing (17,000–29,000 daily exhalations for 12–20 breaths per minute for healthy adults) (Fig. 2).

### Infective quanta

To evaluate indirect exposure risks from vaping, we simplify and adapt the analytic risk model of two articles by Buonanno, Morawska, and Stabile (hereafter BMS) (Buonanno et al. (2020a)), cited in the review (Peng et al. (2021)). BMS have examined the potential SARS-CoV-2 virus transmission in various indoor micro-environments (see also their previous paper Buonanno et al. (2020b) and further updated information in Peng et al. (2021)). BMS develop this model by means of Montecarlo simulations in which variability of droplet emission rates and exposure parameters is described by suitable probability distributions. Our approach is to assume median values for these variables (50 percentiles) of these distributions, similar to their approach in their previous paper (Buonanno et al. (2020b)). This is justified because our aim is to evaluate the risks from indoor COVID-19 transmission from vaping, speaking, and coughing (all episodic or intermittent expirations) in comparison with what can be denoted as a “control case” scenario of risks in a space where the infectious vaper is only rest breathing (a continuous expiration). We are not aiming at providing a full comprehensive risk analysis for each respiratory activity separately under more realistic conditions (something that would justify a full separate study in itself).

BMS consider the notion of an infective “quantum”: the dose of airborne respiratory droplet nuclei necessary to infect 63% of exposed susceptible individuals. They introduce the “quantum emission rate” ${\texttt {ER}}_q$ (emitted quanta per hour) for various respiratory expirations

2 $${ER}_q=\frac{C_v}{C_{RNA\;}C_{PFU}}\times f_{br}V_TC_d,$$

where $c_v$ is the viral load (RNA copies/mL) in the sputum of a SARS-CoV-2-infected person (symptomatic or not), $c_{\text {RNA}}$ is the number of RNA copies per PFU (plaque forming unit) needed to generate infection and $c_{\text {PFU}}$ is quanta-to-PFU conversion parameter, $f_{\text {br}}$ is the number of breaths per hour and $V_T$ the tidal exhaled volume, and ${\mathcal {C}}_d$ is the droplet volume concentration (in $\text {mL}/\text {m}^3$, hence ${\mathcal {C}}_d V_T$ is the total volume of exhaled droplets in mL). BMS define the product “$I{}R= V_T \times f_{\text {br}}$” as an “inhalation rate,” but it can also be used as an exhalation rate expressible in units $\text {m}^3/\text {h}$.

For the infection parameters, BMS consider values that have emerged from recent data: $c_v=10^7$ RNA copies/mL (average in the range $10^3-10^{11}$), $c_{\text {RNA}}= 1.3\times 10^2$ RNA copies/PFU and $c_{\text {PFU}}= 2.1\times 10^2$ PFU/quanta. For the droplet volume concentration, they take as reference an experimental value that incorporated dehydration effects in droplets associated with loud speech (Stadnytskyi et al. 2020) (see Bake et al. 2019 for a review of this type of observational studies of droplet/nuclei emission as well as formation mechanism in the upper and lower respiratory tracts). Then, using experimental data from Morawska et al. (2009) to scale this reference to other respiratory expirations, leading to the following values (in $\text {mL}/\text {m}^3$)

3 $$\begin{aligned} \mathcal {C}_d = 2\times 10^{-2} \text { (loud speech) },\quad 6\times 10^{-3} \text { (normal speech) },\quad 2\times 10^{-3} \text { (rest breathing) }, \end{aligned}$$

### Adaptation of quanta estimations to vaping

In order to fit vaping expirations into these values, we need to make some assumptions on the involved parameters, besides considering the effects on exposure from the time duration of expiratory activities. In particular, we need to evaluate their mean quanta emission rate *only* in the times when they occur and compare with the rates of normal rest breathing (which takes place all the time). To simplify matters, we assume that $c_v, c_{\text {I}}$ and $f_{\text {{(br)}}}$ are largely unaffected by the timing of these expiratory activities. We have then

- **Low-intensity MTL vaping**. A vaper breathes $N_{\text {(tot)}}$ times in (say) 1 h and of these breaths $N_{\text {(vp)}}$ coincide with vaping expirations (puffs), the expression for ${\texttt {ER}}_q$ in (2) must be modified as
  4 $$\begin{aligned} {\texttt {ER}}_{q\text {(vp)}} = \frac{c_v\, f_{\text {br}}}{c_{\text {RNA}}+c_{\text {PFU}}} \left[ \frac{N_{\text {(vp)}}}{N_{\text {(tot)}}}V_{T{\text {(vp)}}} {\mathcal {C}}_{d{\text {(vp)}}} + \left( 1-\frac{N_{\text {(vp)}}}{N_{\text {(tot)}}}\right) V_{T{\text {(br)}}} {\mathcal C}_{d{\text {(br)}}}\right] , \end{aligned}$$
  where $N_{\text {(vp)}} ,N_{\text {(tot)}}$ are the number of vaping puffs and total number of breaths per hour and $V_{T{\text {(br)}}} V_{T{\text {(vp)}}}$ and ${\mathcal {C}}_{d{\text {(vp)}}}, {\mathcal {C}}_{d{\text {(br)}}}$ are the tidal volumes and droplet volume concentration for vaping and rest breathing. For low-intensity MTL vaping, we assume a tidal volume of $V_T=750 \text {cm}^3$ supported by data inferred and discussed in Sussman et al. (2021b), while for droplet volume concentration we assume $\mathcal{C}_d=3\times 10^{-3} \text {mL}/\text {m}^3$, a plausible value denoting emissions slightly above rest breathing but below normal speech in Eq. 3, fitting the “whispered counting” data of Morawska et al. (2009). For the number of breaths, we can take the average values of 160 daily puffs in a 16-h journey (Dautzenberg and Bricard (2015); Cahours and Prasad (2018)) and breathing frequency of $f_{\text {(br)}}=16/\text {min}$ (in the range 12–20), so that $N_{\text {(tot)}}=960 \text {breaths/h}$ and $N_{\text {(vp)}}=10 \text {breaths/h}$.
- **High-intensity DTL vaping**. We assume $V_T=2000 \text {cm}^3$ as an average tidal volume. However, there is ambiguity in inferring a value for droplet volume concentration because of insufficient data on how much the larger tidal volume and deeper inhalation of DTL vaping can modify respiratory droplet numbers and diameters. As mentioned in Section 2 of Sussman et al. (2021b), higher powered devices associated with DTL vaping tend to increase ECA droplet sizes and diameters (Lechasseur et al. (2019); Floyd et al. (2018)) but it is not certain if this applies to respiratory droplets. However, as mentioned in Section 3.3.2 of Sussman et al. (2021b), speech involves droplet generating mechanisms that are distinct from those of breathing (Asadi et al. 2019; Morawska et al. 2009; Johnson et al. 2011), resulting in higher rate of droplet emission even with a tidal volume only slightly larger than the breathing rest value of $400-600 \text {cm}^3$ (Bailey and Hoit (2002); Hoshiko (1965)). Thus, we have two plausible options to account for a higher total volume of exhaled droplets ${\mathcal {V}}_d=V_T {\mathcal {C}}_d$: it may follow simply from a larger $V_T$ with the same value ${\mathcal {C}}_d=3\times 10^{-3} \text {mL}/\text {m}^3$ of low-intensity vaping, or we might assume the larger value of ${\mathcal {C}}_d$ for normal speech in Eq. 3. Instead of choosing one option, we will keep the continuous range of $\mathcal {C}_d=3-6 \times 10^{-3} \text {mL}/\text {m}^3$. Regarding the number of breaths, we can assume the same values as low-intensity vaping: $N_{\text {(tot)}}=960\text {breaths/h}$ and $N_{\text {(vp)}}=10 \text {breaths/h}$.
- **Normal speech**. The equation for ${\texttt {ER}}_q$ in Eq. 2 needs to be modified in a similar way as Eq. 4, replacing the droplet volume concentration $\mathcal {C}_d$ with the value for normal speech in Eq. 3 and we take as tidal volume the value $V_T=600 \text {cm}^3$, roughly $10 \%$ larger than the average rest value (Bailey and Hoit 2002; Hoshiko 1965). To incorporate the timing, we replace $N_{\text {(vp)}}$ with a number count of breaths coinciding with a given percentage of an hour interval spent on continuously speaking in a given indoor environmant. For $5, 10, 20, 30, 40\%$ of the hour (960 total breaths), we have $N_{\text {(sp)}}=48, 96, 192, 288, 384 \text {breaths/h}$.
- **Coughing**. The emission data from coughing in Morawska et al. (2009) is comparable to that of “unmodulated vocalization” (repeating the vowel “aahh”). Hence, we can use Eq. 4 with the value for droplet concentration volume of loud speaking in Eq. 3 as a proxy for coughing, while for coughing tidal volume we have $V_T=1400 \text {cm}^3$ (Gupta et al. (2009)). Assuming a cough every 2 and 3 min, $N_{\text {(vp)}}$ is replaced by $N_{\text {(cf)}}=20, 30$.

Considering the plausible assumptions stated above, we display in Fig. 2 the logarithmic plots of quanta emission rate ${\texttt {ER}}_q$ from an infectious individual as a function of viral load $c_v$, for rest breathing, low- and high-intensity vaping, and speaking for $10 \%, 20 \%, 30 \%$, and $100 \%$ of the time, as well as coughing every 2 and 3 min. The numerical values of ${\texttt {ER}}_q$ in quanta per hour for $c_v =10^7$ RNA copies/mL are

5 $$\begin{aligned} {\texttt {ER}}_{q(\texttt {br})}= & {} 0.3416,\quad {\texttt {ER}}_{q(\texttt {vpL})}=0.3562,\quad {\texttt {ER}}_{q(\texttt {vpH})}=0.3727-0.4139,\nonumber \\ {\texttt {ER}}_{q(\texttt {sp10})}= & {} 0.5063,\quad {\texttt {ER}}_{q(\texttt {sp20})}=0.6610,\quad {\texttt {ER}}_{q(\texttt {sp30})}=0.8158,\nonumber \\ {\texttt {ER}}_{q(\texttt {sp40})}= & {} 0.9705,\quad {\texttt {ER}}_{q(\texttt {cf})} = 1.2637,\quad {\texttt {ER}}_{q(\texttt {sp100})} = 1.8216, \end{aligned}$$

where the symbols br, vpL, vpH, sp10, sp20, sp30, sp40, sp100, and cf respectively denote breathing, vaping low and high intensity, speaking $10, 20, 30, 40, 100\%$ of the hour, and coughing 30 times. In particular, for low- and high-intensity vaping, ${\texttt {ER}}_q$ is very close to the control case of rest breathing (almost indistinguishable for low-intensity vaping), while even speaking $10 \%$ of the hour (6 min) yields a larger ${\texttt {ER}}_q$ value than the upper end of high-intensity vaping. Also, normal speech for a full hour (not uncommon) produces a higher quanta emission than coughing 30 times.

### Exponential dose-response risk model

In order to evaluate a time-dependent risk for expiratory activities that incorporate quanta emission rates and indoor environment variables, BSM consider the “dose response exponential model” given in terms of the the density of the quanta *n*(*t*) in units $\text {quanta}/\text {m}^3$ under the assumption that $n(0)=0$ (no exposure at initial time $t=0$)

6 $$R\;=\;1\;-\;\exp\;\left[-\mathrm{IR}\;\int_0^Tn(t)\;dt\right]=1-\exp\left[-\frac{\mathrm{IR}\;\left[{\mathrm{ER}}_qN\;T\;-\;n(T)\;V\right]}{\mathrm{IVVR}\;\mathrm V}\right],$$

7 $$\begin{aligned} n(t)= & {} \frac{\texttt {ER}_q\, N}{\texttt {IVVR}V}\left[ 1-\exp (-{\texttt {IVVR}}\, t)\right] , \end{aligned}$$

where *V* is the volume ($\text {m}^3$) of the indoor micro-environment, *N* is the number of exposed susceptible individuals, ${\texttt {IR}}$ is the inhalation rate ($\text {m}^3/h$) of these individuals, and ${\texttt {IVVR}}$ is the infectious virus removal rate, which BMS take as the sum of three factors: ${\texttt {IVVR}}={\texttt {AER}}+\kappa +\lambda _0$, where ${\texttt {AER}}$ is the ventilation air exchange rate, $\kappa$ is the particle deposition on surfaces, and $\lambda _0$ is the virus inactivation (all of these quantities given as $h^{-1}$).

We evaluate in the “Results: risk evaluation” section the risk *R* for vaping exhalations and other respiratory activities, aiming at the evaluation of their relative risk with respect to the control state of continuous breathing, assuming a home and restaurant scenarios with natural and mechanical ventilation.

## Results: risk evaluation

To evaluate the risk of exposure to respiratory droplets carried by vaping exhalations in indoor environments, we have adapted in the “Methods: risk model of contagion” section the “dose response exponential model” developed by Bounnano, Morawska, and Stabile (BMS) (Buonanno et al. (2020a))^1^. Specifically, we evaluate equation (6) that defines the risk *R* (as a fraction $<1$) for the value ${\texttt {IR}}=0.96 \text {m}^3/\text {h}$ taken from the previous paper of BMS (Buonanno et al. (2020b)) and justified as a level of physical activity half way between standing and light activity. For the remaining parameters, BSM assume the range ${\texttt {AER}}=0.2-0.5/h$ for natural ventilation and ${\texttt {AER}}=9.6/h$ for a restaurant scenario with mixed ventilation. BMS compute the deposition rate by dividing typical gravitational settling velocity for supermicron particles ($10^{-4} \text {m/s}$) by the height of emission ($1.5 \text {m}$), leading to $\kappa =0.24/h$, while for the viral inactivation they take the measured aerosolized SARS-CoV-2 virus mean life of 1.1 h (Van Doremalen et al. 2020) and even longer periods (Fears et al. (2020)), leading to $\lambda _0=0.63/h$. We consider the following home and restaurant indoor scenarios:

- Home scenario. We assume one infectious vaper and three exposed susceptible family members ($N=3$). Total exposure time $T=12$h. Indoor volume $125 \text {m}^3$ (small $50 \text {m}^2$ apartment with roof height of $2.5 \text {m}$). For natural ventilation, ${\texttt {AER}} = 0.2/\text {h}$ we have ${\texttt {IVVR}}=1.07/\text {h}$.
- Restaurant, natural ventilation with open door. Thirty costumers ($N=30$), total exposure time $T=3$ h. Air exchange rate ${\texttt {AER}} = 0.5/\text {h}$, indoor volume $300 \text {m}^3$ ($100 \text {m}^2$ area with roof height of $3 \text {m}$), results in ${\texttt {IVVR}}=1.37/\text {h}$
- Same restaurant endowed with mechanical ventilation: ${\texttt {AER}} = 9.6/\text {h}$ (taken from Buonanno et al. (2020b)), results in ${\texttt {IVVR}}=10.47/\text {h}$

**Fig. 3:**
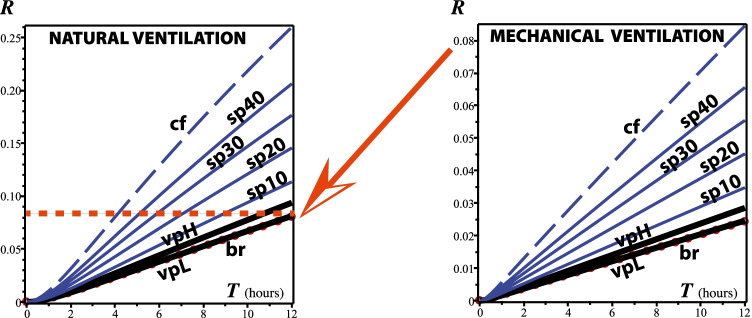
Infection risk in a home environment. The curves display *R* as a function of exposure time *T* from Eq. 6. The abbreviations br, vpL, vpH, sp10, sp20, sp30, sp40, and cf stand for rest breathing; vaping low intensity; vaping high intensity (upper end option); speaking for 10, 20, 30, 40% of time; and coughing. Notice the dramatic reduction of *R* achieved by mechanical ventilation (moderate air exchange rate of $3/\text {h}$). Also, the curves for the risks from vaping (full range of intensities) are practically indistinguishable from that of the case control scenario of rest breathing (red circles). These curves show the same order of magnitude values as those of figure 3 of BMS for their scenarios A-1 and A-2

**Fig. 4:**
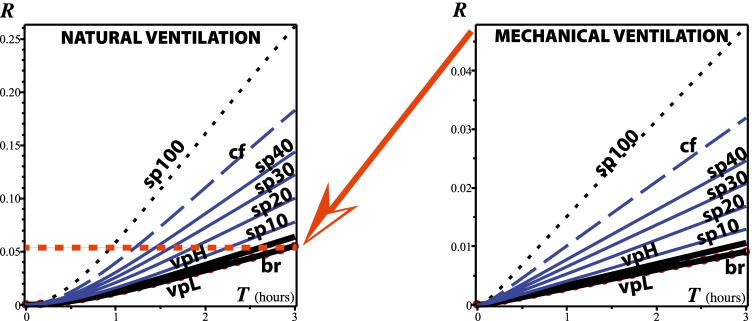
Infection risk in a restaurant. The same abbreviations as in Fig. 2 plus sp100 (speaking 100% of the time, a possible outcome when spending 3 h in a restaurant). As in Fig. 3, mechanical ventilation (air exchange rate $9.6/\text {h}$) achieves a dramatic reduction of *R* and the curves for the risks from vaping are practically indistinguishable from the curve of the control case scenario of rest breathing (red circles). These curves are comparable on orders of magnitude to those of figure 3 of BMS for their scenario C in the time frame of 3 h

**Fig. 5:**
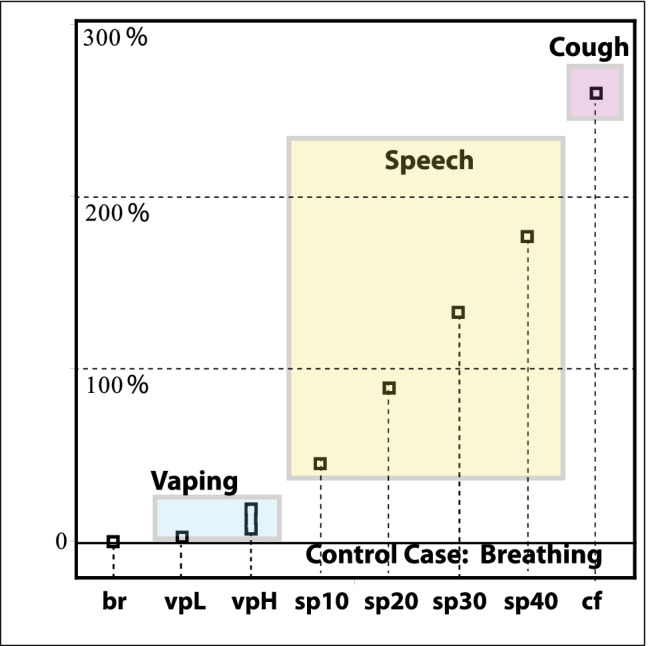
Added percentage risks of expiratory activities with respect to the control case scenario of rest breathing. The percentage values with respect to the control case are low-intensity vaping 1.3% (vpL); high-intensity vaping 5.2–17.7% (vpH); speaking 44% (sp10), 88% (sp20), 132% (sp30), 176% (sp40) for 10%, 20%, 30%, 40% of time; and coughing 259% 30 times per hour (cf). These values were obtained from $(\varepsilon -1)\times 100$ for $\varepsilon$ defined for these expiratory activities by Eqs. 8–10

The infection risk *R* for home and restaurant scenarios is plotted in Figs. 3 and 4 as a function of time for breathing, low- and high-intensity vaping, various percentages of time spent speaking, and coughing every 2 min, considering natural and mechanical ventilation. As expected from the quanta emission rates displayed in Fig. 2, the exposure time of different expirations is a crucial factor in computing *R*. As expected, the risk factor *R* increases with exposure time *T*, displaying an approximately growing linear dependence that keeps the same shape but is is markedly decreased with mechanical ventilation in in each scenario: *R* decreases to one third (25 to 8%) after 12 h exposure in a home environment with air exchange rate of 3/h and one fifth (25 to 5%) after 3 h exposure in a restaurant environment with air exchange rate of 9.6/h. However, the key point is not the absolute values of *R*(*T*) but its comparison for various respiratory activities and the control state of exclusive normal rest breathing. Figures 3 and 4 reveal that exposure to vaping expiration (vaper doing 10 puffs per hour) poses an infection risk to bystanders that is very close to that from the control case scenario. In fact, for low-intensity vaping, the infection risk *R*(*T*) is practically indistinguishable from the control case and even for high-intensity vaping it is well below that from the same person speaking and coughing. Speaking only for $10 \%$ of the time (6 min per hour) already yields a higher infection risk than high-intensity vaping, while speaking $30-40 \%$ of the hour yields up to 4 times the infection risk, which is roughly the values plotted in Fig. 5.

A good inference of the risk from intermittent and episodic expiratory activities (vaping, speaking, coughing) relative to the control case scenario of exclusive rest breathing (a continuous expiration) is furnished by the ratio $R_{\text {(A)}}/R_{\text {(br)}}$, where $A = \text {vp, sp, cf}$ (see Eq. 5). Plotting this ratio from Eqs. 6–7 for every expiratory activity yields near constant curves around the values of the quotients ${\texttt {ER}}_{q\text {(A)}}/{\texttt {ER}}_{q\text {(br)}}$ (see numerical values in Eq. 5). This is not surprising since ${\texttt {ER}}_q$ is the only variable in *R* that characterizes the infectious person (the other variables characterize the indoor micro-environment and the exposed susceptible persons). Hence, given the same indoor micro-environment and same number of susceptible individuals, we consider risks relative to the control case scenario of rest breathing in terms of the ratio of quanta emission. Using Eq. 4, we have

8 $$\begin{aligned} \varepsilon = \frac{{\texttt {ER}}_{q\text {(A)}}}{{\texttt {ER}}_{q\text {(br)}}}=1+ \left( \frac{ {\mathcal {V}}_{d{\text {(A)}}} }{ {\mathcal {V}}_{d{\text {(br)}}} }-1\right) \approx \frac{R_{\text {(A)}}}{R_{\text {(br)}}}, \end{aligned}$$

where ${\mathcal {V}}_{d{\text {(A)}}} = V_{T{\text {(A)}}} {\mathcal {C}}_{d{\text {(A)}}}$ is the total exhaled droplet volume (in mL) for each expiratory activity “*A.*” Since $N_{\text {(br)}}=N_{\text {(tot)}}$, then for a heavy breathing activity in intense aerobic exercise $\varepsilon$ might grow only because of the much larger tidal volume. However, for a truly intermittent expiration like vaping, we have $N_{\text {(vp)}}/N_{\text {(br)}}\ll 1$ and thus $\varepsilon \approx 1$ holds even if we have $\mathcal {V}_{d{\text {(A)}}}/{\mathcal {V}}_{d{\text {(br)}}}\gg 1$ (large exhaled amount of droplets as with the large tidal volumes in extremely intense vaping). For the values of tidal volume and droplet volume concentration we have used the numerical values in Eq. 5, we have the following relative risks

9 $$\begin{aligned} \varepsilon= & {} 1.25 \times \frac{N_{\text {\tiny {(vpL)}}}}{N_{\text {\tiny {(br)}}}}\;\; \text {(low intensity vaping)},\quad \varepsilon =5-11\times \frac{N_{\text {\tiny {(vpH)}}}}{N_{\text {\tiny {(br)}}}}\;\; \text {(high intensity vaping)},\end{aligned}$$

10 $$\begin{aligned} \varepsilon= & {} 3.6 \times \frac{N_{\text {\tiny {(sp)}}}}{N_{\text {\tiny {(br)}}}}\;\; \text {(speaking)},\quad \varepsilon =28\times \frac{N_{\text {\tiny {(cf)}}}}{N_{\text {\tiny {(br)}}}}\;\; \text {(coughing)}, \end{aligned}$$

which provides an intuitive indication of the added exposure risks relative to the control case from the different expiratory activities.

We display in Fig. 5 the numerical values of $\varepsilon$, as an added risk with respect to the control case for various expiratory activities with respect to the continuous presence of risk from rest breathing and under the assumptions we have used. These numbers clearly reflect the effects of the intermittence or duration time of each activity. Under normal vaping conditions (10–15 puffs per hour), the added risk of low-intensity vaping with respect to the control scenario of exclusive rest breathing is of the order of $\sim 1 \%$ (since $\varepsilon -1\sim 10^{-2}$). For high-intensity vaping, it is $\sim 5-17 \%$, given the ambiguity in the range of $\mathcal {V}_d=V_T\mathcal {C}_d$, still it is of the order of $\varepsilon -1\sim 5 \times 10^{-2}-10^{-1}$, also a low added risk since the low value of $N_{\text {(vp)}}/N_{\text {(br)}}$ compensates for the large exhaled tidal volume. Notice that the added risk with respect to the control case grows to $\sim 40 \%$ just for talking for 10% of the time and easily reaches 176% if talking 40% of the time. Coughing is also intermittent, possibly even more intermittent than vaping, but its large amount of exhaled droplets (large factor of 28 in Eq. 10) can offset this effect. For speaking, $\varepsilon$ can be large even if normal speech involves a tidal volume close to rest breathing, but it also involves a much larger amount of time (larger number of breaths in typical conversation).

## Discussion I: comparison with results of BMS

Since we have adapted the risk model developed by BMS to be able to incorporate vaping exhalations in the potential indoor transmission of COVID-19, it is important to compare our results with theirs with the parameters characteristic of their scenarios, bearing in mind as well that we are considering median values of the main parameters (roughly equivalent to 50th percentiles) instead of a statistical distribution to account for their variability. Comparison with BMS also serves a sort of validation of our approach, since the values ${\texttt {ER}}_q$ in quanta per hour for $c_v =10^7$ RNA copies/mL in (5) cover the range of magnitudes as the 50 percentiles values reported for oral breathing and various forms of vocalizing in Table 2 and figure 1a of BMS, as well as the respiratory activities listed in their Table 1.

The first sign that we have obtained analogous risk factors *R* comes from comparing our Figs. 3 and 4 with figure 3 of BMS that also plots *R* vs time, but with the axes inverted: vertical and horizontal axis respectively corresponding time and *R*. Notice that the relation between *R* and time in the scenarios A-1, A-2, and C of BMS depicted in their figure 3, which are roughly comparable to our home and restaurant scenarios, shows the same orders of magnitude as in our Figs. 3 and 4.

**Fig. 6:**
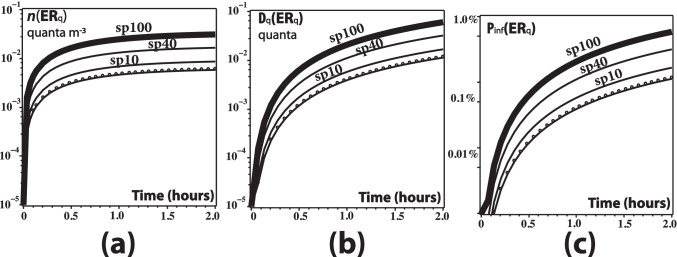
Scenario D of BMS. The panels depict the density of quanta $n(\text {ER}_q)$, the dose of quanta ${\text {D}}_q(\text {ER}_q)$, and probability of infection ${\text {P}}_{\text {{inf}}}$ (in %) from the data of scenario D described by Table 1 of BMS. The curves correspond from bottom to top to breathing, vaping (dashed curve), and speaking 10%, 40% (solid curves) and 100% of the time (thick curve). The latter curve, as expected from this scenario, provides a good approximation to the 50-percentile curves of figure 1 of BMS

We can also approximate the “scenario D” of BMS, defined as an infected subject singing or speaking loudly in an $800 \text {m}^3$ room with healthy subjects listening at a sedentary activity level. Considering the value ${\texttt {ER}}_{q(\texttt {sp100})} = 1.8216$ for quanta emission per hour when speaking 100% of the time, with $N=10$ listeners, ${\texttt {IVVR}}=1.37/\text {h}$, and ${\texttt {IR}}=0.96 \text {m}^3/\text {h}$, we can produce the three graphs displayed in Fig. 6 for the density of quanta *n*(*t*), as well as the dose of quanta ${\texttt {D}}_q$ and infection probability ${\texttt {P}}_{\text {\tiny {inf}}}$ (as percentage), defined as

11 $$\mathrm{D}_{q}(\mathrm{ER}_{q}, T)\int_{0}^{T} n(t)dt, \quad \quad P_{\mathrm{inf}}=1-\mathrm{exp}(-\mathrm{D}_{q})$$

These graphs provide a good approximation to the 50th percentile curves (thick curves) displayed in figure 1 of BMS. However, the interpretation is different: for BMS the spread of values in that figure corresponds to percentile levels representing variation of parameter values in a normal statistical distribution associated with Montecarlo simulations, whereas in our case it corresponds to the median value (50 percentile) for various respiratory activities that would be carried on by the emitter: breathing, vaping (dashed curve), talking 10% (sp10), 40% (sp40), and 100% (sp100) of the time. Notice that (as expected) for the three quantities plotted the best approximation to the 50-percentile curve in the three panels of figure 1 of BMS is the emitter speaking 100% of the time (sp100 thick curve). From the spread of the curves in our Fig. 6, we could argue that assuming mean values of infective parameters the spread of COVID-19 transmission in this scenario would have been greatly diminished if the infected person had spoken (or sung) for only 10% or 40% of the time instead of 100%. Notice also that the curve for vaping is practically indistinguishable with that of breathing.

**Fig. 7:**
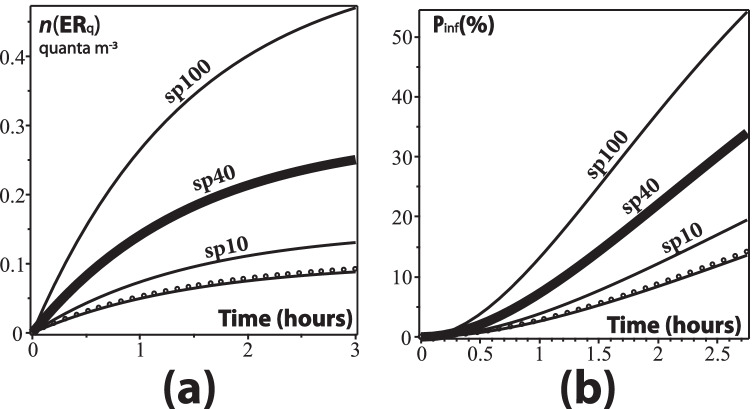
Outbreak at the Guangzhou restaurant. The panels depict density of quanta and probability of infection for the data of the Guangzhou restaurant. The curves correspond from bottom up to breathing, vaping (circles), and speaking 10%, 40%, and 100% of the time (sp10, sp40, sp100). The thick curve (speaking 40% of time) provides the best fit to the curves of figure 4 of BMS

We can also provide a reasonable approximation to the estimation made by BMS of the well-known surge of infections in the Guangzhou restaurant in China. For this case, we take the known data $N=20, V=97 \text {m}^3$ and assume $\text {IR}=0.96$, together with natural ventilation ${\texttt {IVVR}}=0.67/\text {h}$ (values taken from Peng et al. (2021)). As shown in the graphs of *n*(*t*) and ${\text {P}}_{\text {inf}}$ (in percentages) displayed in the two panels of Fig. 7, the best match with the curves of figure 4a of BMS corresponds to the emitter speaking 40% of the time (sp40), which is a reasonable proportion of time for speaking during a meal. As in the previous example of scenario D of BMS, we can also argue that under the assumption of 50-percentile values of infective parameters the spread of COVID-19 would have been greatly reduced if the infected patrons had spoken only 10% of the time (sp10 curve) or remained silent (even if he/she had vaped).

## Discussion II: effects of face mask wearing

In our risk evaluation in a home and restaurant scenarios in the “Results: risk evaluation” section, we did not assume face mask wearing by emitters and receivers of infective quanta. However, this fact has little real-life relevance in our risk evaluation for the home scenario because face masks are rarely worn at home (even under confinement). Assuming that containment measures permit that bars and restaurants remain open, our risk evaluation also remains roughly valid for such venues (if vaping is allowed), even if vaping necessarily requires the vaper to remove the face mask (at least for the brief time lapse of intermittent puffs). In fact, eating and drinking in a restaurant scenario also require face mask removal, which in a convivial atmosphere (with conversations accompany eating and drinking) should involve quanta emissions by mask-free patrons whose duration is likely to exceed the strict time needed to eat and drink. However, it is still necessary to examine exposure risks in hypothetical indoor scenarios in which universal face mask wearing is strictly enforced. In particular, we need to emphasize the effects on those face masks that are usually worn at a community level: surgical masks and/or those made of cotton and other fabrics.

Face masks, in their multiple designs and fiber characteristics (see review in Tcharkhtchi et al. 2020), filter aerosol particles through various physical processes: gravitational sedimentation, inertial impaction, interception, diffusion, and electrostatics, each of which governs and/or becomes dominant in specific ranges of particle sizes, airflow, leaks, and environmental factors according to aerosol filtering theory (see Hinds 1999). The key issue we need to assess is how much cotton and surgical masks protect (in terms of filtering efficiency) their wearers from *inward* emission when they are exposed to *outward* quanta emissions by potentially infectious individuals not wearing face masks (as vapers when vaping). It is especially useful to compare this protection with respect to the one they would get when exposed to emitters who are also masked (i.e., *reciprocal masking*).

Filtering efficiency is high in outward emission for N95 respirators (over 90%) and slightly less so (74%) in surgical masks in human emitters breathing, speaking and coughing (Asadi et al. 2020), with decreasing diameters of filtered droplets, though in these experiments droplet counts excluded ultra-fine droplets below $0.3 \mu$m and leaks were not evaluated. Similar results were obtained in laboratory conditions with a non-biological aerosol, though leaking decreased efficiency in surgical and cotton masks between one-half and two-thirds (Drewnick et al. 2021).

Evidently, face masks also protect their wearers from inward emissions, as revealed by the following two laboratory experiments:

- In Sickbert-Bennett et al. (2020), two human subjects in different body postures, wearing well-fit N95 respirators and surgical masks (tied with stripes or ear loops), were exposed to a non-biological polydisperse aerosol ($d_p=0.02-3.00 \mu \text {m}$) released in a chamber at concentrations between 2000 and 5000 $\text {cm}^{-3}$. Fitted filtration efficiency was above 95% in all N95 respirators, 71.5% for the surgical mask tied with stripes, and 38.1% for the one fit with ear lobes. Efficiency decreased for the latter to 21.2% when the subjects turned their head left or right, showing the effects of leaks.
- In Ueki et al. (2020), a bioaerosol generated by a nebulizer emitted at flow velocity of 2 m/s, simulating a mild cough airflow, was used to examine virus penetration (in terms of virus titer) between two mannequins separated at distances of 50 and 100 cm, in experiments wearing loose and fit N95 respirators and surgical and cotton masks. The filtering efficiency was measured in terms of the detected percentage of virus titer in the receiving mannequin with respect to the titer in the emitting one. For $10^5$ PFU (plaque forming units) when the receiver was wearing different face masks and the emitter was unmasked filtering efficiency was 17%, 47%, 57%, and 79% for the cotton mask, surgical mask, and loose and fit N95 respirators respectively. When both mannequins wore a surgical mask, the filtering efficiency significantly rose to 60%, 71%, 69%, and 92%. Efficiency was about 10% lower for $10^8$ PFU. Virus titers decreased to 45% and 31% when mannequins (both unmasked) were placed at distances of 50 cm and 100 cm with respect to their values at 25 cm separation.

Both laboratory experiments describe, in spite of their idealization, the relatively low protection afforded by cotton and surgical masks to bystanders exposed to either unmasked emitters at close range and/or high droplet concentrations, flow, and virus titer, which are precisely the conditions characteristic of direct exposure that would likely affect bystanders (wearing such masks) placed at the source (close to the mouth) and in the direction of the jet exhaled by an infectious vaper or by someone infectious breathing, talking, or coughing without wearing a mask.

However, as opposed to masked bystanders exposed to mask-less emissions from other respiratory activities, we showed in the “Background II: visualization of the respiratory flow through ECA” section that bystanders close to a vaper in shared indoor spaces can avoid instinctively the spatial zone of direct exposure that is clearly delineated by the visible emission jet exhaled by a vaper. These are evidently very different exposure conditions from those of the experiments described above, as bystanders wearing cotton or surgical masks located outside the exhaled jet would be subjected only to indirect exposure to a very low concentration of submicron droplet nuclei (the so-called aerosols) dispersing through air currents at ambient velocities that depend on the ventilation regime (roughly below 10–20 cm/s with natural ventilation, Matthews et al. 1989; Berlanga et al. 2017). In fact, while SARS-CoV-2 transmission through indirect exposure to these submicron droplets and droplet nuclei (what the WHO and most medical literature denotes as “aerosols”) is an undeniable fact, its scope and reach and its relation to occupancy and ventilation is still controversial (NASEM (2020); Jayaweera et al. (2020); Shiu et al. (2019); Sommerstein et al. (2020)).

In particular, a much decreased airflow in indirect exposure implies a much decreased face velocity $U_f$, the air velocity at the mask surface obtained by dividing the air flow (LT/min) over the mask surface area. For the range of flow of rest breathing 10–25 LT/min, we have $U_f=6-12$ cm/s (see Drewnick et al. 2021), which is qualitatively analogous to characteristic velocities of indoor circulation currents with natural ventilation. Intuitively, under these low-intensity flow conditions, the masks capture more particles (droplets and nuclei) because the permanence time of the latter favors the capture mechanisms that are dominant for the particle diameter $d_p> 0.3 \mu$m: inertial impaction and interception. The dependence of the percentage of filtration efficiency *E* on $U_f$ when these mechanisms are dominant is given by $E\propto [1-\exp (-U_f^{-4/9})]\times 100$ for a broad range of filtering parameters (see equations 9.19 and 9.35 of Hinds (1999)), which for $d_p> 0.3 \mu$m yields $E>90 \%$ (see figures 9.9 and 9.10 of Hinds (1999)). The same arguments should apply to indirect exposure to drifting droplet nuclei from other respiratory activities that are sufficiently small to remain buoyant for long times.

As a consequence, universal wearing of cotton and surgical face masks offers a significantly higher level of protection against indirect exposure to small droplets and nuclei spread once respiratory droplets have evaporated. Evidently, it would be extremely complicated to adapt the relative risk model we have presented in the “Methods: risk model of contagion” section to these conditions, since we would have to re-calculate in terms of the filtering efficiency of the face masks the quanta emission assigned to the control state of breathing emitters, of the comparative emitters talking and coughing, as well as the exposed receivers. However, incorporating this complexity might not be worthwhile after all, given the fact that the added contribution of vaping to the overall respiratory droplet emission from the control state of breathing remains vary small.

## Discussion III: how robust is our relative risk model?

The results of our analysis, as listed in detail in the “Results: risk evaluation” section and Figs. 3, 4, and 5, reveal that vaping expirations (by being intermittent and with low emission rates close to breathing) represent a minor relative risk increase of exposure to SARS-COV-2 transmission with respect to the control state: low-intensity vaping (practiced by 80–90% of vapers) involves a 1% increase of the risk while high-intensity vaping involves an increase of risk of 5–17% (the uncertainty follows from the lack of a precise inference on its droplet emission rate). As a comparison (see Fig. 5), speaking 6–24 min per hour increases the risk by 44–176% and coughing 2 times per minute in an hour by 259%.

How stable are these results under the enormously varied conditions that result from the complexity of the COVD-19 global pandemic? As we argued in the “Results: risk evaluation,” “Discussion I: comparison with results of BMS,” and “Discussion II: effects of face mask wearing” sections, exposure time, face mask wearing, and ventilation introduce significant modifications on the (approximately linear) dependence of infective absolute risk *R* exposure time *T*, though these environmental effects should produce the same modification to all respiratory activities (at the very least there is no evidence to sustain a contrary argument); thus, the comparative relative risks plotted in Fig. 5 should remain roughly constant. This is consistent with the fact that Figs. 3 and 4, which display *R*(*T*) for the various respiratory activities under natural and mechanical ventilation for the same micro-environment conditions (under the parameters used by BMS), show that the slopes for vaping (low and high intensity) in all cases are practically indistinguishable with the slope for the breathing control state.

As argued extensively in the “Background III: viral variants, vaccination, and environmental factors” section, several important factors that modify contagion risks arise as new information on the biological evolution of the SARS-CoV-2 virus emerges, leading to new SARS-CoV-2 variants that are more infectious than the original strain, though this increase in risk can be greatly mitigated by increasing vaccination rates at least in more developed countries. Being an airborne transmitted disease mechanical ventilation is an important modifying factor on the computation of computation of the risk of infection *R*(*T*), but risk estimates might also affect natural ventilation estimates through local variations of temperature and humidity, which (pending on local climate) might also affect indoor spaces. Other factors that might modify contagion risks are atmospheric conditions and air pollution. However, the primary and best known and studied cause of contagion remains airborne transmission in indoor spaces, while research on the effects from environmental factors originating outdoors remaining uncertain and speculative (see discussion in the “Background III: viral variants, vaccination, and environmental factors” section). Nevertheless, it is necessary and important to assess the stability and robustness of our relative model with respect to the all the above mentioned elements of the complexity of COVID-19.

The key argument that sustains the robustness of the model is the hypothesis that all respiratory activities are equally affected by the different factors of COVID-19 complexity that we mentioned above. In other words, there is no evidence that a more infectious variant will generate on infected individuals more (or less) emission of respiratory droplets only for a given respiratory activity (say, speaking) and not in another one. In more technical terms this can be appreciated from Eq. 2 that defines the most basic parameter of our analysis, the quantum emission rate ${\texttt {ER}}_q$ (emitted quanta per hour). A more infectious SARS-CoV-2 variant should change only the infective parameters in this equation:

- $c_v$, the viral load (in RNA copies/mL) in the sputum of an infected person,
- $c_{\text {\tiny {RNA}}}$, the number of RNA copies per PFU needed to generate infection,
- $c_{\text {\tiny {PFU}}}$, the quanta-to-PFU conversion parameter,

but should not have any effect on the parameters specific to respiratory activities: $f_{\text {{br}}}$ (number of breaths per hour) and $V_T$ (tidal exhaled volume), nor on ${\mathcal {C}}_d$, the droplet volume concentration (in $\text {mL}/\text {m}^3$), or ${\mathcal {C}}_d V_T$, the total volume of exhaled droplets in mL. We remark that there is an immense individual variation in these parameters, with some individuals being recognized as “super spreaders”, but there is no evidence that that different SARS-CoV-2 variants have any influence on these parameters, which largely depend on physiological characteristics if individuals and of mechanisms for generating respiratory droplets.

A more infectious variant should imply either more virus loaded respiratory particles in a given emission or the same number of such particles but each carrying a heavier viral load (as seems to be the case with the Delta variant, see the “Background III: viral variants, vaccination, and environmental factors” section). Another possibility regarding the efficient transmissibility of the Omicron variant would be from its enhanced ability to infect evading SARS-CoV-2 immunity caused by either vaccination or past infection (Koslow (2022)). In all cases, these possibilities imply modifications of the numerical values of the infective parameters, but not of the parameters of the respiratory activities. This in turn would modify the numerical evaluation of ${\texttt {ER}}_q$ in Eq. 5 but in a way that is proportionate to the parameters of the respiratory activities. This would modify the absolute contagion risk in Eqs. 6–7 that explicitly depends on the numerical values of ${\texttt {ER}}_q$ for the different respiratory activities. However, these modification would not affect the relative risk defined with respect to the control case of exclusive rest breathing: $R_{\text {{(A)}}}/R_{\text {{(br)}}}$, where $A = \text {vp, sp, cf}$ defines the respiratory activity (see Eq. 5), so that plotting Eqs. 6–7 will yield near constant curves around the values of the quotients ${\texttt {ER}}_{q\text {{(A)}}}/{\texttt {ER}}_{q\text {{(br)}}}$. The near equivalence of the ratio of quanta emission to the ratio of contagion risk associated to the control state should yield the same values in Eq. 8 and thus we would obtain basically the same results as displayed in Fig. 5.

Whether our hypothesis of independence of infective parameters on the respiratory activities holds or not should be verified empirically. The effects from environmental factors, such as temperature, humidity, air pollution and atmospheric dynamics, should also be independent of the respiratory activities. As more information and data emerge from the new variants, it might be possible to design the appropriate experiments for testing this hypothesis. However, as long as there is no contrary evidence, we believe that this is a valid working hypothesis and that the results we have obtained should be robust with respect to the complexity that characterizes the COVID-19 pandemic.

## IV: safety considerations

As mentioned in the introduction, available evidence suggests that aerial COVID-19 contagion has occurred through a wide spectrum of environmental conditions between direct exposure to “droplets” (droplets with $d_p> 5 \mu$m) and indirect exposure to “aerosols” (small droplets and droplet nuclei $d_p< 5 \mu$m) (see NASEM 2020; Jayaweera et al. 2020; Shiu et al. 2019; Sommerstein et al. 2020 and further upgrade in Peng et al. 2021). However, the distinction between “droplets” and “aerosols” has practically no effect for the risk evaluation we have undertaken, since bystanders easily avoid direct exposure by virtue of the visibility of vaping and our goal has been the evaluation of relative risks of respiratory activities in comparison with a well defined control state of pure breathing.

### Safety measures

As shown in the “Background II: visualization of the respiratory flow through ECA” section, vaping expirations potentially carrying the SARS-CoV-2 virus are visible (as opposed to other respiratory activities). Besides the evident psychological dimension of this flow visualization, there are safety implications: vapers and those surrounding them have a clear, instinctive and immediate delineation of the flow’s horizontal and vertical distance reach and spreading direction along the exhaled jet. From the outcomes of the hydrodynamical analysis carried in Sussman et al. (2021b), we can recommend as a basic safety measure to avoid direct exposure (irrespective of face mask wearing) by keeping a 2-m distance away from the vaper (when vaping) in the direction of the visible jet. However, notice that exhalation ranges above 2 m are unusual, as 80–90% of vapers use low powered devices whose exhaled jets reach well below 2 m (and typically exhaling with a 30° downward angle, see Sussman et al. (2021b)). In other directions away from the jet (even at close distance), the exposure is indirect, but nevertheless as a safety measure it is prudent to maintain 2 m of separation in all directions from anyone vaping when not wearing a face mask. Notice that these recommended safety measures coincide with the standard social separation recommendations adopted worldwide (Hsiang et al. 2020).

#### Face masks

In computing exposure risks in the “Results: risk evaluation” section, we did not consider face mask wearing. This is justified because face masks are not usually worn in a home scenario. Even in a restaurant or bar scenario, patrons are likely to remain mask-less for extended periods because masks must be removed for eating and drinking (as with vaping). As we discuss in the “Discussion II: effects of face mask wearing” section, face masks of common usage (surgical and cotton) afford limited protection to bystanders wearing them subjected to direct exposure to respiratory droplets from mask-free emitters. However, once outside the direct exposure zone (visible and delineated for vaping), bystanders wearing common usage face masks would be facing practically the same risks from indirect exposure as in spaces in which no vaping took place.

#### Lockdown vs opening

Risk assessments are essential to provide evidence based support for preventive and mitigating policies that have been proposed and enacted worldwide (see review Hsiang et al. 2020). These assessments are sensitive to the wide variety of rapidly changing pandemic conditions and scenarios. High levels of severity characterized by frequent contagion rates can be addressed by lockdowns contemplating different levels and stages of home confinement. Under these conditions the risk assessment for the home scenario that we presented is particularly relevant, as a large number of vapers and smokers become home bound for a range of large periods. As we argue in Sussman et al. (2021a), the risk assessment undertaken in the present paper provides valuable information for safety policies in this scenario: low-intensity vaping only produces a minuscule ($\sim 1 \%$) extra contagion risk with respect to the control case scenario of continuous breathing. Safety interventions should consider that abstention from vaping would not produce a noticeable safety improvement, but could generate an undesired level of stress and anxiety under long term confinement. High-intensity vaping produces a higher increase of relative risk, but still well below speaking and coughing. Notice that face masks are seldom worn in home bound scenarios of family clusters.

#### SARS-CoV-2 variants and vaccination

Evidently, safety considerations must vary following the biological evolution of the SARS-CoV-2 virus, as well as on the response to it by means of vaccination process and other non-pharmaceutical preventive and containment measures. More infectious new SARS-CoV-2 variants necessarily require improving safety considerations, as absolute risks increase for all respiratory activities, including vaping, but this assessment depends on whether exposed individuals or the vaper have been vaccinated. Safety considerations under these constantly varying conditions can become complicated and strongly dependent on vaccination rates and face mask usage which vary for different countries. However, our safety considerations that come form our main result still holds: in comparison with vocalizing and coughing, vaping represents a small increase of contagion risk in indoor spaces with respect to the control state of exclusive breathing.

## Limitations

The COVID-19 pandemic is a global dynamical phenomenon of high complexity and multiple regional variations, different responses and attitudes, whose study and understanding requires a dedicated multidisciplinary approach. For these reasons, it is practically impossible for any risk model to be applicable everywhere and at all times, but this complexity does not invalidate simplified or idealized risk models that cover only specific issues of the pandemic. While this article has presented a consistent model of COVID-19 contagion risks in two indoor scenarios for vaping, as a respiratory activity comparable to other such activities (breathing, vocalizing, coughing), it is important to describe its main limitations.

Lack of empiric data. Given the lack of experimental and observational data on respiratory droplets carried by exhaled ECA, we had to consider as basic input for the risk model the data inferred in Sussman et al. (2021b) on the basis of theoretical speculation from the physical and chemical properties of ECA and extrapolation from available data on other expiratory activities (cigarette smoking and mouth breathing with a mouthpiece) that can serve as reasonable proxies for vaping. Evidently, the present paper inherits another important limitations of Sussman et al. (2021b): the oversimplification of vaping styles by classifying a complex usage pattern into two categories, “low” and “high” intensity vaping, which cannot capture the full range and scope of individual vaping habits.

Oversimplification of infective parameters and individual variability. The rates of emitted droplets inferred for vaping are rough average estimates gathered from outcomes reported in breathing studies (see Table 2 of in Sussman et al. (2021b)), involving a wide variety of subjects, including both healthy and individuals affected by respiratory conditions (not by SARS-CoV-2). Also, we did not considered the small minority of outlier individuals who are super spreaders emitting significantly larger numbers of droplets (Asadi et al. 2019). Also, the data on infective SARS-CoV-2 parameters gathered by BMS that we use in the “Methods: risk model of contagion” section is also subjected to uncertainties that they specifically recognize. In fact, numerous aspects associated with the spreading and infection details of the SARS-CoV-2 virus remain uncertain and subject to large (often unexplained) individual and environmental variability (a good summary of these uncertainties is found in Klompas et al. (2020); Morawska and Milton (2020); Morawska and Cao (2020); NASEM (2020); and Peng et al. (2021)). However, in order to be able to model a possible (previously unexplored) route of droplet transmission and possible infection, it is necessary and unavoidable to simplify this complexity and lack of data to obtain plausible order of magnitude estimates that can be verified once empiric evidence is available.

Oversimplification of the risk model. The adapted BMS risk model that we presented in the “Methods: risk model of contagion” section is also simplified (see details in section 2.1.4 of BMS). While it fulfills our aim of providing a rough comparative estimation of relative risks with respect to the control case of continuous rest breathing, we do recognize its limitations: the risks are evaluated for a single vaper in highly idealized micro-environments, assuming constant infection parameters and inhalation rates (which BMS also assume), ignoring as well probability distributions of the quanta emission rates that convey individual variation on infection susceptibility and other parameters (which the model of BMS does incorporate). A more elaborate and complete approach should include a more robust methodology to quantify exposure risks to intermittent and sporadic sources, as for example in Nazaroff (2004); Ai et al. (2019), or a more complete description of environmental parameters as in Peng et al. (2021). This task is left for a future analysis.

SARS-CoV-2 variants and vaccination and environmental factors. In the computation of the quantum emission rate, we used numerical values of infective parameters that were valid in 2020, before the massive vaccination effort and when there were fewer variants. However, these parameters are certainly varying in more infectious variants of the SARS-CoV-2 virus that have emerged. Incorporating the dynamical complexity of the COVID-19 pandemic would necessarily require evaluating absolute risks for each emerging SARS-CoV-2 variant, considering vaccination rates at each jurisdiction and a number of other factors and elements that we did not include in our analysis. However, as we argued in the “Discussion III: how robust is our relative risk model?” section, we assumed as working hypothesis that this multifactorial dynamic evolution characterizing the pandemic should equally affect all respiratory activities. Therefore, while exposure and absolute risks associated to each respiratory activity will necessarily vary along a complicated pattern, the relative risks of respiratory activities with respect to a control state defined by exclusive rest breathing should remain relatively stable.

Environmental factors. We did not consider environmental factors, some of which can influence indoors transmission of the virus (temperature and humidity), while others (air pollution and atmospheric dynamics) some originate outdoors but might also affect indoor spaces. Air pollution affects global health conditions (in general) and thus it might play a role also on the spread and evolution of the pandemic. However, as we argued in the “Background III: viral variants, vaccination, and environmental factors” section, contagion risks in large open outdoor spaces are way below the indoor airborne transmission, the main cause of COVID-19 contagion. The influence of these environmental factors on COVID-19 contagion is constrained by the complexity of the wide variation of conditions that occur in air dynamics between outdoor and indoor spaces. Also, research on how and to what degree these factors influence the spread of COVID-19 is still in its early stages, and thus results from many studies are still uncertain and speculative, but further advancing this research might be very useful to promote environmental measures to handle future challenges to global health.

## Conclusion

We have presented in this paper a risk analysis of COVID-19 contagion through direct and indirect exposure to the SARS-CoV-2 virus potentially carried by respiratory droplets and droplet nuclei that would be carried by ECA (e-cigarette aerosol) exhaled by vapers in shared indoor spaces (home and restaurant scenarios). This risk analysis is based on suitable adaptations of the risk model presented by BMS (see Buonanno et al. 2020a, b) that incorporates experimental data on SARS-COV-2 infective quanta: we consider vaping expirations characterized by the respiratory parameters inferred in Sussman et al. (2021b), and we also considered the quantitative effects of exposure from the characteristic duration times of vaping and of other expiratory activities (breathing, vocalizing, and coughing). In particular, given the fact that breathing is a continuous (and unavoidable) expiratory activity, we considered the rate of infective quanta of pure breathing (without vocalizing, coughing, or vaping) as a “control state” that serves as reference to evaluate comparative risks for the rest of the inspiratory activities. To complement this risk analysis, we also discussed the visibility of vaping expirations (the “Background II: visualization of the respiratory flow through ECA” section), the usage of face masks in the indoor scenarios under consideration (the “Discussion II: effects of face mask wearing” section), the possible effects in risk evaluation of new variants of the SARS-CoV-2 virus, and vaccination rates, as well as environmental factors (the “Background III: viral variants, vaccination, and environmental factors” section).

Vaping expirations represent a minimal increase of risk with respect to continuous breathing in home and restaurant scenarios with natural and mechanical ventilation (1% and 5–17% for low- and high-intensity vaping). Visibility of vaping expirations is protective, as it allows avoidance of the high risk of direct exposure to droplets and droplet nuclei potentially carrying the SARS-CoV-2 virus. Those sharing indoor spaces with vapers do not require extra safety interventions besides those already recommended for the general population: wearing face masks and keeping a separation distance of 1.5–2 m to avoid direct exposure. Setting aside harms from environmental tobacco smoke unrelated to COVID-19, these recommendations should also apply to sharing an indoor space with a smoker.

The rapidly varying complexity of the COVID-19 global pandemic makes it very challenging to elaborate a risk model without making simplifying assumptions that may allow for results that can be valid in a wide range of conditions. In this paper, we considered as a working hypothesis the assumption that all modifying factors (virus variants, vaccination rates, and environmental condition) act on equal form on all respiratory activities. This means that (for example) a new infectious variant like Omicron is bound to produce the same extra contagion risk for breathing, vocalizing, coughing, and vaping. While this hypothesis needs to be tested, as far as we are aware, there is no contrary evidence that would show that a given modifying factor acts differently for different respiratory activities. By taking this hypothesis as valid, we can state that while absolute contagion risks for every individual respiratory activity are necessarily dependent on widely varying specific local conditions, the contagion risk of these activities with respect to the control state should remain relatively stable in all conditions. Notice that the rapid evolution of the pandemic places severe limitations on the validity of the results from all types of studies. Nevertheless, the idealized risk model that we have presented can be a useful first step to advance in our understanding of risks of contagion from airborne transmission of the SARS-CoV-2 virus and pandemics that might occur in the future.

## Data Availability

Not applicable.

## Abbreviations

ECA: E-cigarette aerosol
COVID-19: Coronavirus disease 2019
SARS-CoV-2: Severe acute respiratory syndrome coronavirus 2
BMS: Bounnano, Morawska, and Stabile
MTL: Mouth to lung (vaping style)
DTL: Direct to lung (vaping style)
RNA: Ribonucleic acid
PFU: Plaque forming units
br: Breathing
vp: Vaping
vpL: Vaping low intensity
vpH: Vaping high intensity
sp10: Speaking 10% of time
sp20: Speaking 20% of time
sp30: Speaking 30% of time
sp40: Speaking 40% of time
sp100: Speaking 100% of time
cf: Coughing

## Units

LT: Liters
mL: Milliliters
m: Meters
cm: Centimeters
$$\mu$$m: Micrometers
s: Seconds
h: Hour

## Variables

$$V_T$$: Tidal volume
$N_p$: Number of particles
$d_p$: Diameter of particles
$n_p$: Number of particles
$$U_0$$: Exhalation velocity
$\alpha$: Diameter to wavelength ratio
*L*: Cross section distance within the aerosol
$$\lambda$$: Light wavelength
*I*: Beam intensity
$I_0$: Incident beam intensity
$$\sigma _c$$: Extinction coefficient
$Q_c$: Scattering extinction coefficient
$\bar{d}$: Mean particle diameter
$\ell$: Mean free path between scattering events
${\texttt {ER}}_q$: Quanta emission rate
$c_v$: Viral load (RNA copies per mL)
$c_{\text {{RNA}}}$: Number of RNA copies per PFU
$c_{\text {{PFU}}}$: Quanta to PFU conversion parameter
$f_{\text {{br}}}$: Number of breaths per hour
$C_d$: Droplet volume concentration ($\text {mL/m}^3$)
$${\texttt {IR}}$$: Inhalation rate
$N_{\text {{(tot)}}}$: Total number of breaths per hour
$N_{\text {{(vp)}}}$: Number of breaths/puffs per hour for vaping
$N_{\text {{(sp)}}}$: Number of breaths per hour while speaking
${\mathcal {V}}_d$: Total volume of droplets
*R*: Risk for expiratory activities
*T*: Exposure time
*n*: Density of quanta
*N*: Number of exposed susceptible individuals
*V*: Volume of indoor space
${\texttt {IVVR}}$: Infective virus removal rate
${\texttt {AER}}$: Air ventilation exchange rate (per hour)
$\kappa$: Particle deposition on surfaces (per hour)
$\lambda _0$: Virus inactivation (per hour)
${\text {D}_q}$: Dose of quanta (quanta)
${\text {P}}_{\text {{inf}}}$: Probability of infection (percentage)
$$U_f$$: Face velocity
*E*: Efficiency of filtration

## Footnotes

1 We do not assume face mask wearing in this subsection. Effects of face masks are discussed in the “Discussion II: effects of face mask wearing” section.
